# Hyperinsulinemic Hypoglycemia Associated with a Ca_V_1.2 Variant with Mixed Gain- and Loss-of-Function Effects

**DOI:** 10.3390/ijms23158097

**Published:** 2022-07-22

**Authors:** Sebastian Kummer, Susanne Rinné, Gunnar Seemann, Nadine Bachmann, Katherine Timothy, Paul S. Thornton, Frank Pillekamp, Ertan Mayatepek, Carsten Bergmann, Thomas Meissner, Niels Decher

**Affiliations:** 1 Department of General Pediatrics, Neonatology and Pediatric Cardiology, University Children’s Hospital, 40225 Duesseldorf, Germany; frank.pillekamp@med.uni-duesseldorf.de (F.P.); mayatepek@med.uni-duesseldorf.de (E.M.); thomas.meissner@med.uni-duesseldorf.de (T.M.); 2 Institute of Physiology and Pathophysiology, Vegetative Physiology, University of Marburg, 35043 Marburg, Germany; rinne@staff.uni-marburg.de; 3 Institute for Experimental Cardiovascular Medicine, University Heart Center Freiburg—Bad Krozingen, Medical Center—University of Freiburg, 79085 Freiburg im Breisgau, Germany; gunnar.seemann@uniklinik-freiburg.de; 4 Medizinische Genetik Mainz, Limbach Genetics, 55128 Mainz, Germany; nadine.bachmann@medgen-mainz.de (N.B.); carsten.bergmann@medgen-mainz.de (C.B.); 5 Children’s Hospital Boston, Harvard Medical School, Boston, MA 02115, USA; katherinewtimothy@gmail.com; 6 Division of Endocrinology and Diabetes, Cook Children’s Medical Center, Fort Worth, TX 76104, USA; paul.thornton@cookchildrens.org

**Keywords:** calcium channel, hyperinsulinism, *CACNA1C*

## Abstract

The voltage-dependent L-type calcium channel isoform Ca_V_1.2 is critically involved in many physiological processes, e.g., in cardiac action potential formation, electromechanical coupling and regulation of insulin secretion by beta cells. Gain-of-function mutations in the calcium voltage-gated channel subunit alpha 1 C (*CACNA1C*) gene, encoding the Ca_V_1.2 α_1_-subunit, cause Timothy syndrome (TS), a multisystemic disorder that includes autism spectrum disorders and long QT (LQT) syndrome. Strikingly, TS patients frequently suffer from hypoglycemia of yet unproven origin. Using next-generation sequencing, we identified a novel heterozygous *CACNA1C* mutation in a patient with congenital hyperinsulinism (CHI) and associated hypoglycemic episodes. We characterized the electrophysiological phenotype of the mutated channel using voltage-clamp recordings and in silico action potential modeling experiments. The identified Ca_V_1.2^L566P^ mutation causes a mixed electrophysiological phenotype of gain- and loss-of-function effects. In silico action potential modeling supports that this mixed electrophysiological phenotype leads to a tissue-specific impact on beta cells compared to cardiomyocytes. Thus, *CACNA1C* variants may be associated with non-syndromic hyperinsulinemic hypoglycemia without long-QT syndrome, explained by very specific electrophysiological properties of the mutated channel. We discuss different biochemical characteristics and clinical impacts of hypoglycemia in the context of *CACNA1C* variants and show that these may be associated with significant morbidity for Timothy Syndrome patients. Our findings underline that the potential of hypoglycemia warrants careful attention in patients with *CACNA1C* variants, and such variants should be included in the differential diagnosis of non-syndromic congenital hyperinsulinism.

## 1. Introduction

The voltage-dependent L-type calcium channel isoform Ca_V_1.2 is critically involved in cardiac electrophysiology and the regulation of beta cell insulin secretion. Gain-of-function mutations in the calcium voltage-gated channel subunit alpha 1 C (*CACNA1C*) gene, encoding the α_1_ subunit of Ca_V_1.2, are known to cause Timothy syndrome (TS) by inducing intracellular Ca^2+^ overload due to a loss of voltage-dependent channel inactivation. This results in severe cardiac arrhythmia as the cardinal symptom, often leading to death in early childhood. Besides further syndromic features like syndactyly, immune deficiency, cognitive abnormalities and autism, intermittent hypoglycemia of yet unproven origin was reported in 36% of patients [1]. Ca_V_ channels play a key role in regulating the insulin secretion of pancreatic beta cells in response to blood glucose levels [2]. In particular, Ca_V_1.2 accounts for ~45–60% of calcium influx and is the predominant mediator of first-phase insulin secretion in mice [3,4]. In human beta cells, Ca_V_1.2 and Ca_V_1.3 are the main drivers of insulin secretion [5]. However, human beta cells are more heterogenous regarding Ca_V_1 subtype currents, leading to some degree of functional interference between different Ca_V_1 subunits and other non-L-type calcium channels [5].

Given the critical role of Ca_V_1.2 in beta cell signaling and the marked frequency of hypoglycemia in patients with TS, *CACNA1C* is a promising candidate gene for screening patients with congenital hyperinsulinism (CHI). CHI is the most frequent cause of persistent hypoglycemia in early childhood, with an estimated incidence of 1/28,000 to 1/50,000 in non-consanguineous populations [6,7,8]. CHI is caused by inappropriate insulin secretion, leading to recurrent episodes of hyperinsulinemic hypoglycemia, posing a significant risk for impaired neurodevelopment [9,10]. To date, multiple genes have been reported to cause monosymptomatic CHI, and several additional syndromic forms of CHI have been described [11]. However, depending on the subtype of the disease, extensive sequencing strategies cannot assign a clear genetic diagnosis in up to 53% of patients with CHI [12,13]. Next-generation sequencing (NGS) opens the possibility of simultaneously analyzing a multitude of genes to screen for novel candidates in presumably genetic diseases, such as CHI [14,15]. However, so far, patients with monosymptomatic CHI due to mutations in *CACNA1C* have not yet been reported.

Here, we present a patient suffering from CHI in which targeted NGS revealed a heterozygous variant in *CACNA1C*. We provide comprehensive evidence to explain the specific functional consequences of beta cell and cardiomyocyte electrophysiology corresponding to the clinical phenotype. These findings suggest that genetic Ca_V_1.2 dysregulation may cause monosymptomatic CHI without the clinical characteristics of TS.

## 2. Results

### 2.1. Patient History and Metabolic Phenotype

We present a now 17 year-old girl suffering from congenital hyperinsulinism (CHI). She was born in Croatia at term to healthy, non-consanguineous parents (German mother and Croatian father). At 8 months of age, she had a generalized seizure in the early morning after her second overnight fast in life. In the hospital, recurrent hypoglycemia was noted. Metabolic evaluation of hypoglycemia showed a typical profile of hyperinsulinemic hypoglycemia (glucose 37 mg/dL [2 mmol/L], insulin 6.1 mU/L, betahydroxybutyrate [BHB] 0.15 mmol/L, plasma glucose increased from 40 to 79 mg/dL [2.22 to 4.38 mmol/L] on glucagon administration). Diazoxide was started and increased up to 13.2 mg/kg/d which improved glycemic control and better fasting tolerance, but did not allow safe overnight fasting. At the age of 9 months, a single dose of octreotide (3.1 µg/kg) was added daily at bedtime to allow overnight fasting.

Later, octreotide was given four times a day, and then at the age of 3 years and 3 months changed to long-acting somatostatin analogue lanreotide (somatuline autogel^®^, Ipsen Pharma, Munich, Germany), which was started with 60 mg every 4 weeks and then increased progressively over the following months up to 120 mg in the age of 3 years and 9 months. Diazoxide was continued with 5–7.5 mg/kg/d (higher dosage was not accepted due to pronounced hypertrichosis). Thereby, glycemic control was improved and overnight fasting for 11 h was possible with blood glucose >60 mg/dL (aged 4 years), but she still exhibited intermittent hypoglycemia <60 mg/dL several times a week.

At the age of eight years, a glucose tolerance test was performed, showing reactive hyperinsulinemic hypoglycemia 2 h after oral administration of 1.75 g glucose/kg body weight (plasma glucose 46 mg/dL [2.55 mmol/L], insulin 10.3 mU/L, BHB 0.1 mmol/L, free fatty acids [FFA] 1.0 mmol/L).

At the age of 9 years, intermittent hyperglycemia of up to 200 mg/dL (11.1 mmol/L) developed, still alternating with recurrent hypoglycemia, so treatment was continued. At the same time, a formal fasting study showed fasting tolerance of 13 h before hypoglycemia occurred (plasma glucose 48 mg/dL [2.66 mmol/L], insulin 4.6 mU/L, BHB 0.1 mmol/L, FFA 0.49 mmol/L). The oral glucose tolerance test (1.75 g glucose per kg body weight) now showed marked hyperglycemia in the diabetic range after glucose loading (0 min: glucose 65 mg/dL [3.6 mmol/L], insulin 8.0 mU/L; 60 min: glucose 302 mg/dL [16.76 mmol/L], insulin 25.3 mU/L; 120 min: glucose 241 mg/dL [13.37 mmol/L], insulin 23.7 mU/L). In the formal evaluation, fasting tolerance reached 16 h before hypoglycemia occurred (aged 10 years, 7 months).

In the most recent evaluation at the age of 16 years, diazoxide was recently discontinued; there were less frequent and less severe hypoglycemia, but still requiring continuation of lanreotide treatment.

### 2.2. Cardiac Evaluation of the Index Patient

Transthoracic echocardiography was normal at all times. However, electrocardiography revealed QTc intervals in the normal or upper normal range in the majority of examinations. Borderline QTc prolongation slightly >450 ms was seen only twice—once spontaneously and once even during a nifedipine trial, although nifedipine is expected to shorten the QTc time. The QTc intervals at different ages were 456 ms at seven years, 432 ms at ten years, 466 ms under 0.43 mg/kg nifedipine and 422 ms after stopping nifedipine at ten years, 430 ms at eleven years, and 424 ms at twelve years. During spiroergometry at 10 years of age, QTc was 420 ms normal. At no time were there signs of cardiac arrhythmias; in particular, there was no evidence for tachyarrhythmias/torsade de pointes/syncope. In summary, these criteria result in a Schwartz Score of ≤1, indicating low probability of long QT syndrome (LQTS) [16].

### 2.3. Genetic Analysis and Therapeutic Trial with Nifedipine

**Figure 1:**
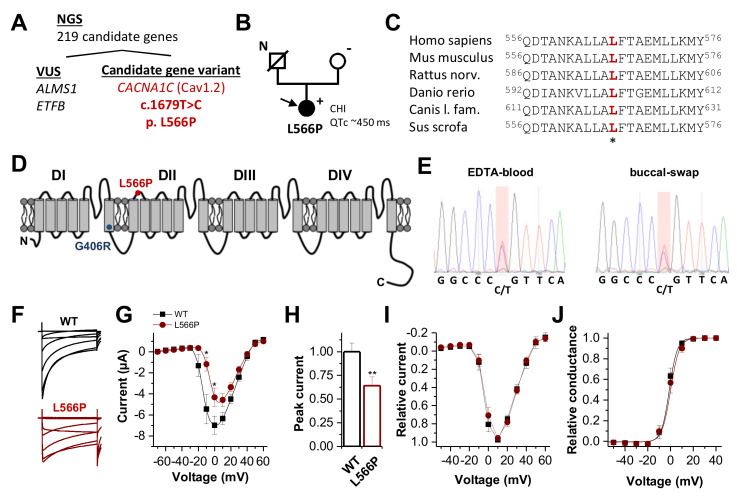
Ca_V_1.2 channel topology, localization and conservation of the Ca_V_1.2^L566P^ mutation identified by WES and electrophysiological characterization. (**A**) NGS panel sequencing of 219 genes associated with familial hyperinsulinism or related disorders of the glucose metabolism variants filtered out four variants of uncertain significance (VUS) and one candidate gene variant in CACNA1C. (**B**) Pedigree of the family. N, no genomic data available; +, heterozygous carrier of c.1679T > C; −, absence of the variant. The filled circle and arrow indicate the diseased index patient. CHI, congenital hyperinsulinism. (**C**) Partial amino acid sequence alignment of Ca_V_1.2, illustrating the conservation of the respective leucine residues among the different orthologues. (**D**) Ca_V_1.2 channel topology. DI to DIV indicate the four calcium channel domains, each consisting of six transmembrane segments and one pore-forming domain. The localization of the channel variants Ca_V_1.2^L566P^ and Ca_V_1.2^G406R^ is indicated as red and blue dots, respectively. (**E**) Electropherogram of the mutation carrier confirming the CACNA1C variant by Sanger sequencing of DNA isolated from EDTA blood and buccal swabs of the patient. (**F**) Representative current traces of Ca_V_1.2^WT^ (black) and Ca_V_1.2^L566P^ (red) recorded from a holding potential of −80 mV, with voltage steps of 1 s duration ranging from −70 to +60 mV in 10 mV increments. The interval between the steps was 30 s. (**G**) Current–voltage relationship of wild-type Ca_V_1.2 (black) (*n* = 19) and Ca_V_1.2^L566P^ (red) (*n* = 14) channels recorded from three independent batches of oocytes. (**H**) Analysis of the relative peak current amplitudes normalized to wild-type Ca_V_1.2. (**I**) Relative current–voltage relationships of wild-type Ca_V_1.2 (black) (*n* = 10) and Ca_V_1.2^L566P^ (red) (*n* = 10) normalized to the peak current. In this analysis, only recordings with peak current amplitudes <5 µA were considered. (**J**) Conductance–voltage relationship of Ca_V_1.2^WT^ and Ca_V_1.2^L566P^ (red). **, *p* < 0.01; *, *p* < 0.05.

Next-generation sequencing (NGS) of 219 genes associated with familial hyperinsulinism or related disorders of glucose metabolism (e.g., monogenic diabetes, disorders of glycogen/fatty acid/ketone metabolism) revealed one candidate gene variant in *CACNA1C* (Figure 1A,B). Here we identified a heterozygous nucleotide exchange from T to C at position c.1679 in exon 13 of the *CACNA1C* gene (c.1679T > C) (NM_000719.6), leading to a leucine-to-proline exchange at position 566 (p.Leu566Pro) (Figure 1A). The leucine residue is highly conserved (Figure 1C) and located in the S2–S3 linker of the second domain (Figure 1D). This *CACNA1C* variant was not reported in the literature (HGMD, PubMed) or in any polymorphism database (e.g., gnomAD, 1000 genomes). All pathogenicity prediction tools (PPTs) that we utilized predicted that the amino acid exchange should be pathogenic. NGS revealed two heterozygous variants of unknown significance in genes known to be relevant for disturbed glucose regulation (Figure 1A and Table S2). However, these variants either do not sufficiently explain the phenotype of the patient or bioinformatics assessment (PPTs, allele frequency) does not support pathogenicity (Table S2). The *CACNA1C* variant was confirmed by Sanger sequencing of DNA isolated from the EDTA blood and buccal swabs of the patient (Figure 1E). As such, there is no indication of low-grade mosaicism restricted to particular tissues. Segregation analysis of the patient’s mother revealed no indication of the detected variant. The DNA of the patient’s father was not available, as he had passed away years before.

A therapeutic trial with calcium channel antagonist nifedipine did not lead to a significant increase in plasma glucose or decrease in plasma insulin after a single administration of 0.2 mg/kg nifedipine, nor an improvement in hypoglycemia rate or fasting tolerance during 7 days of administration (1 mg/kg/d of extended-release nifedipine). Because of minor QTc prolongation during nifedipine treatment and lack of glycemic response, nifedipine was stopped after 7 days.

### 2.4. Loss-of-Function by Altered Current Amplitudes of Ca_V_1.2^L566P^ Mutant Channels

Wild-type Ca_V_1.2 or Ca_V_1.2^L566P^ mutant channels were co-expressed with their β_2_b and α_2_δ subunits in *Xenopus* oocytes. Figure 1F illustrates representative recordings of Ca_V_1.2-encoded currents with typical fast activation and a rapid onset of inactivation. On a first glance Ca_V_1.2^L566P^ mutants revealed a slowing of the inactivation kinetics (Figure 1F) and reduced current amplitudes (Figure 1F,G), with a significant reduction in the peak current densities of about 40% (Figure 1H). The bell-shaped current–voltage relationship (*IV*) of Ca_V_1.2^L566P^ appears to be shifted by about +10 mV (Figure 1G). However, this apparent effect is not present when wild-type Ca_V_1.2 and Ca_V_1.2^L566P^ mutants are studied with similar current amplitudes and a peak current amplitude of a maximal 5 µA (Figure 1I). Consistently, the conductance–voltage relationship (*GV*) is not altered for wild-type and Ca_V_1.2^L566P^ mutant channels when constructs are compared with similar current amplitudes (Figure 1J).

### 2.5. Gain-of-Function Caused by Slowing the Voltage-Dependent Inactivation of Ca_V_1.2^L566P^

**Figure 2:**
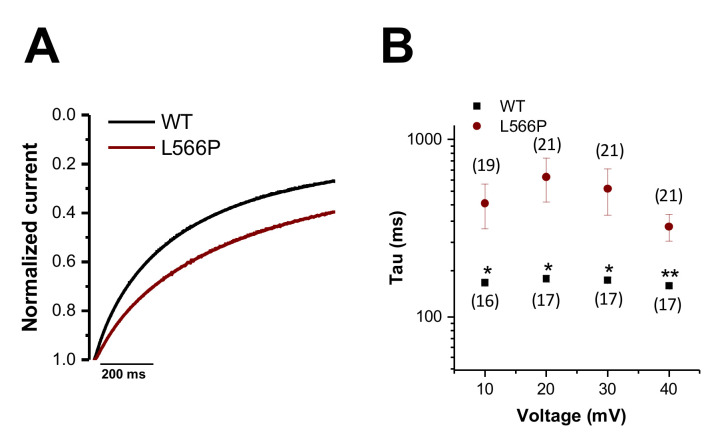
*Kinetics of the voltage-dependent inactivation of Ca_V_1.2^WT^ and Ca_V_1.2^L566P^.* (**A**) Averaged Ca_V_1.2^WT^ (black) and Ca_V_1.2^L566P^ (red) current traces at +20 mV recorded from a holding potential of −80 mV, with a voltage step of 1 s duration to +20 mV (*n* = 17 and *n* = 21, respectively). (**B**) Time constants of inactivation for Ca_V_1.2^WT^ (black) (*n* = 16–17) or Ca_V_1.2^L566P^ (red) (*n* = 19–21) at different potentials for the first 200 ms of inactivation derived by a mono-exponential fit. **, *p* < 0.01; *, *p* < 0.05.

As Ba^2+^ was used in the bath solution as a charge carrier, the channels do not inactivate in a Ca^2+^-dependent manner. Thus, the relatively slow inactivation kinetics observed in these voltage-clamp recordings reflect the voltage-dependent inactivation of the channels. Since the Ba^2+^ encoded currents of Ca_V_1.2^L566P^ appear to inactivate slower and thus less extensively (Figure 2A), we compared the kinetics of voltage-dependent inactivation by analyzing the first 200 ms of current decay (Figure 2A,B). Figure 2A illustrates the averaged wild-type and Ca_V_1.2^L566P^ current traces at +20 mV, in which the slowing of inactivation becomes evident. In summary, we observed a significant slowing of the inactivation kinetics in the voltage range of +10 to +40 mV (Figure 2B), which functionally reflects a gain-of-function.

### 2.6. Gain-of-Function by a Positive Shift in the Voltage Dependence of Inactivation and Reduced Steady-State Inactivation of Ca_V_1.2^L566P^

**Figure 3:**
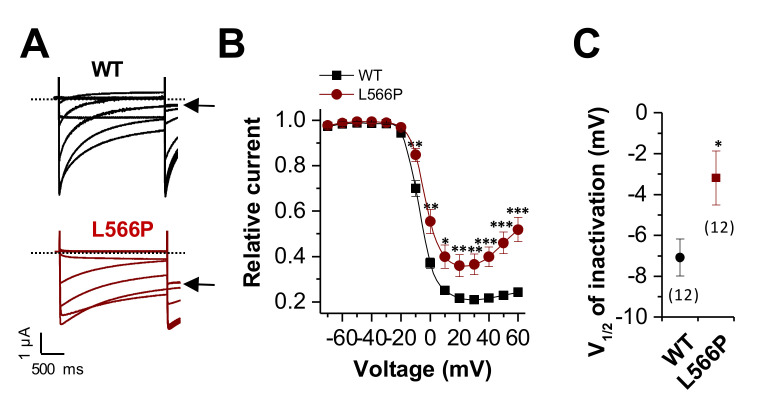
*Reduced and shifted voltage dependence of inactivation of Ca_V_1.2^L566P^.* (**A**) Representative current traces of Ca_V_1.2^WT^ (black) or Ca_V_1.2^L566P^ (red) recorded from a holding potential of −80 mV. Voltage was stepped to potentials ranging from −70 to +60 mV in 10 mV increments for 2 s, followed by a step to +10 mV. The sweep time interval was 30 s. (**B**) Steady-state inactivation curves derived by plotting the prepulse potential against the initial current recorded at the test pulse at 10 mV. (**C**) V_1/2_ of inactivation of Ca_V_1.2^WT^ (black) or Ca_V_1.2^L566P^ (red) derived by fitting the data from (**B**) to a Boltzmann equation. The number of experiments is indicated. ***, *p* < 0.001; **, *p* < 0.01; *, *p* < 0.05.

Next, we analyzed the voltage dependence of inactivation, analyzing the steady state of inactivation, using a pre-pulse protocol (Figure 3A). As illustrated in Figure 3A, wild-type Ca_V_1.2 shows almost complete inactivation (*black arrow*). In comparison, Ca_V_1.2^L566P^ had strongly reduced steady-state inactivation (Figure 3A, *black arrow*). Thus, as described for Timothy and LQT8 syndrome, the mutant has a defect in the voltage dependence of inactivation, with reduced steady-state inactivation (Figure 3B). In addition, we observed a small but significant positive shift in the voltage dependence (V_1/2_) of inactivation of about +4 mV (Figure 3C). Both the reduced steady-state inactivation and the positive shift in the voltage dependence of inactivation reflect a gain-of-function.

Thus, the Ca_V_1.2^L566P^ causes a mixed phenotype with a loss-of-function, due to reduced peak current amplitudes, and a diverse set of gain-of-function effects by an impaired voltage-dependent inactivation, including a slowing of the rate, an altered voltage dependence and a reduced steady-state of inactivation.

### 2.7. In Silico Modeling the Effects of Ca_V_1.2^G406R^ and Ca_V_1.2^L566P^ on Cardiac Action Potentials

**Figure 4:**
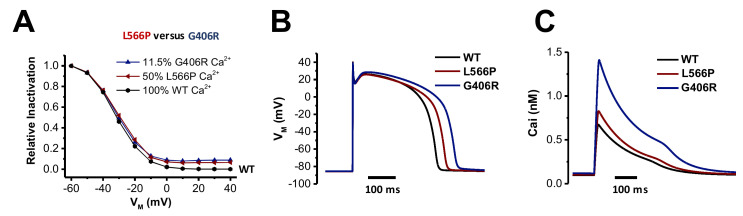
*Stronger APD prolongation for the Timothy mutation Ca_V_1.2^G406R^ compared to Ca_V_1.2^L566P^.* (**A**) Model of the voltage dependence of inactivation of calcium currents in cardiomyocytes for heterozygous patients using the ten Tusscher and Panfilov variants for Ca_V_1.2^G406R^ (blue) and Ca_V_1.2^L566P^ (red), calculated with Ca^2+^ as charge carrier. Indicted are the percentage of mutant Ca_V_1.2 transcripts in the hearts of heterozygous patients. (**B**) In silico ventricular action potential for Ca_V_1.2^WT^ (black) or the heterozygous mutations Ca_V_1.2^G406R^ (blue) and Ca_V_1.2^L566P^ (red). (**C**) Corresponding intracellular Ca^2+^ concentrations during ventricular action potential.

Next, we investigated the impact of the two different mutations affecting mostly Ca_V_1.2 inactivation (Ca_V_1.2^G406R^ and Ca_V_1.2^L566P^) on the action potential (AP) and calcium content using an in silico approach. These experiments might demonstrate the differential effects that the two Ca_V_1.2 mutations might have on electrophysiological properties. Timothy syndrome (Ca_V_1.2^G406R^) is characterized by an almost complete loss of voltage-dependent inactivation. As this mutation in classical Timothy syndrome is only found in exon 8A, only 11.5% of Ca_V_1.2 channels express this mutation in the hearts of heterozygous patients [1]. The novel Ca_V_1.2^L566P^ mutation described here shows a much less pronounced loss of inactivation, but the mutation is not located in an alternatively spliced exon; thus, in heterozygous patients, 50% of the channels are affected by the mutation. We integrated the voltage-clamp data of both mutations into the human ventricular myocyte model of ten Tusscher and Panfilov [17] by adjusting the steady-state voltage-dependent inactivation, the respective inactivation time constants and the current amplitudes (see Materials and Methods). We added 11.5% (Ca_V_1.2^G406R^) or 50% (Ca_V_1.2^L566P^) mutant currents to the respective fractions of wild-type (WT) current to represent the heterozygous state and the expression of the mutants. Although the recorded steady-state inactivation of both mutants appears quite different, the simulated heterozygous behavior is very similar up to a voltage of −10 mV (Figure 4A). AP duration (APD_90_, Figure 4B) was significantly prolonged more in Ca_V_1.2^G406R^ (77 ms) compared to Ca_V_1.2^L566P^ (20 ms), paralleled by a higher resting calcium content in the sarcoplasmic reticulum (SR; Ca_V_1.2^WT^ = 3.1 μM; Ca_V_1.2^L566P^ = 3.2 μM; Ca_V_1.2^L566P^ = 4.4 μM). As a large fraction of the AP is above −10 mV, the larger loss of inactivation of Ca_V_1.2^G406R^ leads to a stronger influx of calcium via Ca_V_1.2 compared to Ca_V_1.2^WT^ or Ca_V_1.2^L566P^ (Figure 4C). This might explain both the more pronounced prolongation of the QT interval and the higher arrhythmogenic potential of Ca_V_1.2^G406R^ observed in patients with Timothy syndrome, as a higher SR calcium content increases the risk for spontaneous calcium releases and thus after depolarizations.

### 2.8. Modeling the Effects of Ca_V_1.2^G406R^ and Ca_V_1.2^L566P^ on Pancreatic Beta Cells

**Figure 5:**
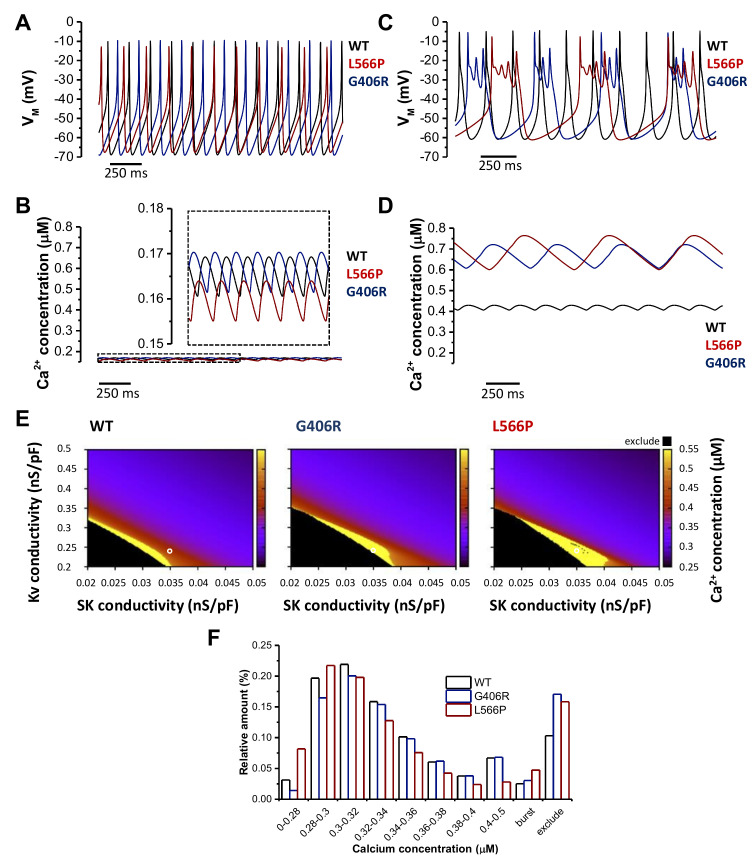
Computational model of the electrical activity of pancreatic beta cells for the wild-type model and the two heterozygous mutations Ca_V_1.2^L566P^ and Ca_V_1.2^G406R^. (**A**) Basal transmembrane voltage (V_M_) characteristics in the Riz et al. model of pancreatic beta cells of Ca_V_1.2^WT^ (black) and for heterozygous Ca_V_1.2^G406R^ (blue) and Ca_V_1.2^L566P^ (red) mutations. (**B**) Corresponding basal intracellular Ca^2+^ concentration oscillations. (**C**) Bursting pattern calculated using the Riz et al. model for Ca_V_1.2^WT^ (black) and heterozygous Ca_V_1.2^G406R^ (blue) and Ca_V_1.2^L566P^ (red) mutations. (**D**) Corresponding Ca^2+^ concentration course during bursting. (**E**) Heat map representing Ca^2+^ concentration in dependence of Kv channel conductivity g_Kv_ and SK channel conductivity g_SK_ for the three investigated cases. Yellow areas represent the burst mode, black area cells that do not repolarize anymore with high calcium concentrations (thus excluded from the evaluation), and the remaining basal mode. (**F**) Relative distribution of the intracellular Ca^2+^ concentrations for Ca_V_1.2^WT^ and for the heterozygous Ca_V_1.2^G406R^ and Ca_V_1.2^L566P^ mutations calculated from the heat maps in (**E**).

As Ca_V_1.2 is also expressed in pancreatic beta cells and the patient with the Ca_V_1.2^L566P^ mutation suffered from hyperinsulinemic hypoglycemia, we further investigated the effects of the two mutations (Ca_V_1.2^G406R^ and Ca_V_1.2^L566P^) on electrophysiology in an in silico model of human beta cells by Riz et al. [18]. It is important to remember that the inflowing calcium via the voltage-gated calcium channel Ca_V_1.2 triggers the release of secretory granules containing insulin. The model of Riz et al. does not model the release of insulin but of electrophysiology and calcium (as markers for insulin secretion). As for the human ventricular myocyte model, we adjusted the steady-state voltage-dependent inactivation, the respective inactivation time constants and the current amplitudes to reflect the measured data (see Material and Methods). In addition, 11.5% (Ca_V_1.2^G406R^) and 50% (Ca_V_1.2^L566P^) mutant currents were added to respective fractions of wild-type (WT) currents to represent the heterozygous state. For the basal mode of beta cell action (also called the spiking mode), we used the same parameters as in the original publication by Riz et al. [18]. Figure 5A,B illustrate the results of the simulations for WT, Ca_V_1.2^G406R^, and Ca_V_1.2^L566P^ for transmembrane voltage and calcium concentration, respectively. The AP shape and spontaneous depolarization rate differ for all three cases only marginally in this basal mode. Beta cells harboring the Ca_V_1.2^L566P^ mutation are predicted to beat 8% slower. In addition, the intracellular calcium concentration is quite similar in all three cases, with the Ca_V_1.2^L566P^ carrying beta cells having a 3% lower basal level.

Next, we in silico modeled the burst mode of beta cells. To induce membrane oscillations, the maximum SK channel conductivity (g_SK_) was reduced from 0.1 nS/pF to 0.035 nS/pF, and the maximum voltage-gated potassium (Kv) channel conductivity (g_Kv_) was reduced from 1 nS/pF to 0.24 nS/pF (see conditions indicated by a white circle in Figure 5E).

While the WT beta cell did not burst with these parameters (Figure 5C), both mutant cells burst with the Ca_V_1.2^L566P^ cell having a lower frequency but a longer burst phase. This led to differences in the intracellular calcium concentrations (Figure 5D). For WT, the mean concentration increased from 0.166 µM to 0.42 µM, for Ca_V_1.2^G406R^ from 0.167 µM to 0.667 µM, and for Ca_V_1.2^L566P^ from 0.16 µM to 0.688 µM. Figure 5E,F show the result of a sensitivity analysis in order to illustrate the higher probability of the mutations drifting into the burst mode (yellow area in Figure 5E) when varying g_SK_ and g_Kv_. The yellow areas in Figure 5E illustrate the conditions under which the cells are in burst mode are larger for the mutations compared to the WT. In addition, these are shifted more toward the upper right corner (in which the parameters for the basal mode are), implying that less pronounced changes in g_SK_ and/or g_Kv_ already lead to the burst mode for the mutant compared to the WT cells, indicating a higher chance of hyperinsulinemic hypoglycemia. The black areas illustrate the electrophysiological situation in which the cells do not repolarize anymore (and thus we excluded them from the evaluation) and under these conditions, the calcium concentrations are raised even more. The effects of the three cases are quantified in Figure 5F. Both the excluded area and the burst area are larger for the mutations, with Ca_V_1.2^L566P^ having a significantly larger burst area. These data indicate that the Ca_V_1.2^L566P^ mutation can lead to more severe hyperinsulinemic hypoglycemia compared to Ca_V_1.2^G406R^. Interesting to note, though, is that Ca_V_1.2^L566P^ seems to have a lower calcium concentration in the basal mode (larger relative amount in bins with lower calcium concentrations), explaining why the hyperinsulinemic hypoglycemia may not appear constantly in our patient.

## 3. Discussion

Voltage-gated calcium channels link intracellular energy metabolism via ATP-induced membrane depolarization to a precisely regulated calcium influx into the cell, which is the key inductor of insulin exocytosis [2,19]. Ca_V_1.2 is the most important Ca_V_ isoform in human beta cells, providing the majority of the calcium influx responsible for insulin secretion [4]. Thus, the *CACNA1C* gene is a promising candidate for screening in patients with dysregulated insulin secretion, e.g., congenital hyperinsulinism (CHI). While there have been two reports about CHI associated with *CACNA1D* variants, coding for the Ca_V_ α_1_ subunit of the Ca_V_1.3 calcium channel, which is also expressed in human pancreatic beta cell [20,21], to our knowledge, *CACNA1C*-associated cases of CHI have not yet been reported. However, there are descriptions that hypoglycemia may remarkably occur in 36% of patients with *CACNA1C*-associated Timothy syndrome [1], further suggesting a role of *CACNA1C* for human insulin secretion and blood glucose regulation. Similar observations have been made for KCNQ1-related long-QT syndrome, which has already been shown to be associated with insulin-mediated hypoglycemia [22].

Here, we present a patient suffering from non-syndromic CHI, bearing a Ca_V_1.2^L566P^ missense variant at a highly conserved position in *CACNA1C* that has not yet been described in the literature or any databases. All bioinformatic pathogenicity prediction tools predicted the variant to be pathogenic. As we could not further validate pathogenicity based on segregation analysis, as the patient’s father had died years ago, we decided to do further functional analyses to assess its biological relevance. Electrophysiological studies of the Ca_V_1.2^L566P^ variant expressed in *Xenopus* oocytes revealed very specific differences to Ca_V_1.2^WT^ but also to the Ca_V_1.2^G406R^ channel variants, with a partial loss-of-function by altered current amplitudes and a partial gain-of-function by impaired voltage-dependent inactivation, including a slowing of the rate of inactivation, an altered voltage dependence of inactivation and a reduced steady-state of inactivation. To explain the marked phenotypic differences of our patient with Timothy syndrome, we aimed to assess the cell type-specific consequences of these electrophysiological properties by comprehensive in silico modeling of cardiomyocytes and beta cells. We show that the Ca_V_1.2^L566P^ variant has only minor effects on the cardiac action potential in an in silico model—much less than the classical Ca_V_1.2^G406R^ Timothy syndrome variant. In contrast, the Ca_V_1.2^L566P^ variant led to markedly increased calcium flux via Ca_V_1.2 channels in the in silico model of beta cells, while the Ca_V_1.2^G406R^ variant had less pronounced effects in the pancreatic beta cell model. These findings are in perfect agreement with the beta-cell dominated phenotype associated with the Ca_V_1.2^L566P^ mutation, while the phenotype of the Ca_V_1.2^G406R^ mutation is dominated by the long QT syndrome/cardiomyocyte pathophysiology. The effects of the Ca_V_1.2^L566P^ mutation on beta cell oscillations might be so pronounced in comparison to that of Ca_V_1.2^G406R^ (1) due to a stronger transcriptional expression of the mutation, as it affects all pancreatic Ca_V_1.2 transcripts and not only those harboring alternative exon 8 and (2) the mixed gain-of-function and loss-of-function phenotypes. Strikingly, the changes in inactivation induced oscillations only in the in silico model when we concomitantly reduced the current amplitudes, as observed in our voltage-clamp recordings. The fact that the Ca_V_1.2^L566P^ mutation does not cause a pronounced LQT prolongation as Ca_V_1.2^G406R^ (note that the QTc interval of our patient was yet ‘on the long side’) might also be attributed to this mixed gain-of-function and loss-of-function phenotype combined with the completely different electrophysiology of cardiomyocytes. The cardiac action potential has an overshoot and membrane potentials remain over 0 mV for quite some time [23], while human pancreatic ß-cells do not reach these potentials at all [24]. In addition, one does not observe membrane oscillations in cardiomyocytes physiologically, but rather a long action potential with a plateau phase [23]. During this relatively steady depolarized plateau phase, the reduced inactivation of Ca_V_1.2^L566P^ might be just counterbalanced by the decreased current amplitudes of the mutant, leaving the net calcium flux and the plateau phase largely unaffected. Thus, electrophysiological differences of the mutations (affecting 11.5% versus 50% of transcripts in heterozygous patients; shifts in the voltage dependence or the kinetics of inactivation; altered current amplitudes or mixed gain- and loss-of-function effects), together with the different types of action potentials, are likely to induce tissue-specific phenotypes.

Another explanation for the phenotypic variation of the Ca_V_1.2^L556P^ and Ca_V_1.2^G406R^ variants may be the specific location in the protein, as *CACNA1C* transcripts underlie highly variable and tissue-specific splicing processes [25,26]. The Ca_V_1.2^G406R^ Timothy syndrome variant most frequently affects an alternatively spliced transcript of Exon 8 (Exon 8A), which is only weakly expressed in pancreatic tissue but highly expressed in heart and blood vessels [1]. This further explains why patients with classical Timothy syndrome have a much less pronounced pancreatic/metabolic phenotype and an exaggerated cardiac phenotype than the patient presented here. Moreover, for some Timothy syndrome patients, phenotypic variations were described as a consequence of somatic mosaicism [27,28]. However, in our case, sequencing revealed a 50% mutational burden in DNA from leukocytes and cheek swabs, corresponding to a heterozygous mutation. This widely excludes somatic mosaicism as a potential cause of why our patient presents a phenotype different from classical Timothy syndrome.

Regarding therapeutic management, after obtaining the genetic test results, it was obvious to attempt treatment with nifedipine to counteract the increased calcium flux via the mutated Ca_V_1.2 variant. Nifedipine has been used for the treatment of congenital hyperinsulinism in the past [29,30], this approach turned out to be generally ineffective [31], and thus is no longer commonly used for other forms of CHI. Here, even though we found in vitro that the Ca_V_1.2^L566P^ mutation is still nifedipine sensitive (data not shown), this did not lead to any significant clinical improvement. It remains unclear whether this may be explained by specific properties of the channel variant or if this just reflects the widespread unresponsiveness that has been already described for other types of CHI as well. However, we did not increase the dose beyond 1 mg/kg/d, so we cannot finally exclude a therapeutic response to higher doses of, e.g., 2 mg/kg/d that were used by others [29,32]. On the other hand, given that the Ca_V_1.2^L566P^ mutation induces a mixed gain-of-function and loss-of-function electrophysiological phenotype, one would not expect a Ca_V_1.2 blocker to restore the wild-type function of the channel.

To further extend the knowledge about the metabolic consequences of *CACNA1C* variants, we performed a retrospective analysis of TS patients reporting hypoglycemia. Five patients/families responded to one of the authors (K.T.) inquiry to provide medical records for the hypoglycemia episodes (clinical hallmarks outlined in Table S3). However, despite hundreds of pages of retrospective medical records, these do not provide sufficient metabolic data to track the occurrence of hypoglycemia to a specific/consistent metabolic phenotype. In three of five patients, no metabolic parameter except glucose was determined during hypoglycemia, so a more specific metabolic footprint of hypoglycemia is not available in these patients. One had ‘40 mg/dL of urinary ketones’ documented in the context of hypoglycemia; it is unclear which kind of determination was used. However, this represents at least a significant amount of ketosis, which does not support insulin-mediated hypoglycemia. Another patient had only ‘traces’ of urinary ketones detectable after hypoglycemia, so an insulin-mediated mechanism seems possible. Although these data do not allow firm conclusions, they are more suggestive of a mixed phenotype, including ketotic, thus not insulin-mediated hypoglycemia, and hypoketotic (thus potentially hyperinsulinemic) hypoglycemia and not a very specific metabolic footprint of hypoglycemia in TS patients in general. We then identified one TS child who, after a history of sporadic hypoglycemia during gastroenteritis, underwent endocrine/metabolic workup at the age of two years. Tests showed normal fasting tolerance with appropriate ketotic response to fasting, normal cortisol and growth hormone response, but reactive hypoglycemia on the oral glucose tolerance test (Table S4). These as-of-yet inconsistent observations warrant further examination, e.g., careful biochemical evaluation of spontaneous hypoglycemia in children with TS to provide further evidence of whether there is a more consistent biochemical footprint of hypoglycemia in TS than we could conclude from the retrospective anecdotal data analyzed here. However, we can indeed conclude from the retrospective data that hypoglycemia is a major cause of morbidity and even mortality in Timothy syndrome, as there tragically were two fatalities in the context of hypoglycemia, possibly due to hypoglycemia-triggered cardiac deterioration/arrhythmia. Thus, performing provocative hypoglycemia/fasting studies for metabolic characterization of TS patients needs to be carefully limited to those with immediate consequences for therapeutic management and is probably not justified for study purposes only because of the reported casualties associated with hypoglycemia.

A limitation of our work is the lack of in vitro data validating the consequences of the Ca_V_1.2^L566P^ variant specifically in beta cells. While we made vigorous attempts to establish a Ca_V_1.2^L566P^ bearing beta cell line, these experiments proved exceptionally difficult. First, we did not succeed in establishing a transfection model because of inefficient transfection, lack of stable alignment with the other channel subunits, and achieving a knockdown of the wild-type background at the same time in appropriate proportions to approximate the heterozygous state in our patient. Furthermore, we were unable to achieve CRISPR-Cas9 knock-in of the Ca_V_1.2^L566P^ variant in a beta cell line because of the location of the variant quite distant to potential CRISPR-Cas restriction sites. Thus, we cannot prove the hyperinsulinism phenotype directly in beta cells bearing a heterozygous Ca_V_1.2^L566P^ variant. However, the strength of our work is that we show very comprehensive electrophysiological data revealing a pattern very distinctively different from Ca_V_1.2^WT^ and Ca_V_1.2^G406R^ Timothy syndrome variants. We are confident that our mechanistic workup still provides compelling evidence of the pathogenicity of the Ca_V_1.2^L566P^ mutation. Second, precise electrophysiological data allow for a robust transfer into established in silico models of beta cells and cardiomyocytes, which nicely mirror clinical observations. Although we can report only one patient, our data may help us understand the in vivo biological consequences of altered calcium channel physiology in humans. Furthermore, the work presented here might serve as an example of how comprehensive electrophysiology and in silico modeling might contribute to estimating the pathogenicity of putative genetic variants in channel genes.

In summary, we present a patient with monosymptomatic CHI and a mutation in the Ca_V_1.2 calcium channel. This Ca_V_1.2 mutation causes a significantly altered channel electrophysiology, leading to a mixed gain-of-function and loss-of-function phenotype primarily affecting the membrane oscillations of beta cells, which are essential for insulin secretion. From these data, we conclude that this variant is causative for CHI in this patient and propose that this gene is a new CHI-causing gene that should be included in the genetic differential diagnosis of CHI. In contrast, hypoglycemia episodes in Timothy syndrome seem biochemically more diverse and warrant further research and attention.

## 4. Materials and Methods

### 4.1. Index Patient

The index patient was identified from a group of patients with CHI at the University Children’s Hospital, Duesseldorf, where all clinical examinations were performed. The diagnosis of CHI was based on common clinical criteria (see the patient description for more details). Laboratory evaluation was performed in the clinical routine laboratory with standardized clinical assays and cardiologic evaluations using routine facilities/techniques in the hospital. Electrocardiography was analyzed by an experienced pediatric cardiologist. QT intervals were frequency corrected using Bazett’s formula and given in ms. Genetic testing was performed within the frame of a registered clinical study (German Clinical Trials Register ID DRKS00006874) and approved by the Ethics Committee of the Medical Faculty of the Heinrich-Heine University Duesseldorf (ID 4790R). Written informed consent was obtained for inclusion in the study and for being part of this publication.

### 4.2. Mutation Analysis in the Index Patient

In brief, we utilized a customized sequence capture library that targeted exons and an additional 35 bp of flanking intronic sequence of 219 genes known or hypothesized to cause monosymptomatic or syndromic CHI or other forms of monogenic glucose dysregulation (Table S1). Genomic DNA was fragmented, and the coding exons of the analyzed genes, as well as the corresponding exon–intron boundaries, were enriched using the Roche/NimbleGen sequence capture approach (NimbleGen, Madison, WI, USA), amplified and sequenced simultaneously by Illumina NGS sequencing-by-synthesis technology using an Illumina HiSeq 1500 system. A mean target coverage of 624× on the HiSeq system with about 99% of the target regions covered with at least 20×. NGS data analysis was performed using bioinformatic analysis tools as well as JSI Medical Systems software (version 4.1.2, J.S.I. Medical Systems, Ettenheim, Germany). Identified variants and indels were filtered against external and internal databases and filtered depending on their allele frequency, focusing on rare variants with a minor allele frequency (MAF) of 1% or less. Variant classification followed ACMG/AMP guidelines [33] and ACGS Best Practice Guidelines for Variant Classification in Rare Disease 2020 (https://www.semanticscholar.org/paper/ACGS-Best-Practice-Guidelines-for-Variant-in-Rare-Ellard-Baple/7923ccd6fdd28c82e48f05f5117d8bd261d180d9, accessed on 27 June 2022) taking into account the current literature and database situation as well as clinical data/laboratory parameters and bioinformatics prediction programs. High coverage enabled copy number variation (CNV) analysis. Potential copy number alterations (CNA) were initially identified by VarScan on mapped reads. Thereby, coverage of every target region of the sample was internally normalized and compared versus the normalized control data of other samples of the same run by VarScan copy number mode and standard settings. Putatively pathogenic variants were validated by conventional Sanger sequencing.

### 4.3. Calcium Channel Expression and Electrophysiological Characterization in Xenopus Oocytes Xenopus

Oocytes were prepared as previously described [34]. Isolated oocytes were stored at 18 °C in ND96 solution containing in mM: NaCl 96, KCl 2, CaCl_2_ 1.8, MgCl_2_ 1, HEPES 5; pH 7.4 with NaOH, supplemented with Na-pyruvate (275 mg/L), theophylline (90 mg/L) and gentamicin (50 mg/L). Ca_V_1.2 wild-type and mutant channels were subcloned in pBS, linearized with NotI and cRNA was synthesized using a mMessage mMaschine T7 Transcription Kit (Ambion). Ca_V_β_2_b was subcloned in pSPORT, linearized with NotI and cRNA was prepared as mentioned above. Ca_V_α_2_δ long was subcloned in pSP65, linearized with SalI and cRNA was made with SP6 polymerase [1]. Stage IV and V oocytes were injected with 11 ng of human Ca_V_1.2 wild-type or mutant and 1.35 ng rabbit Ca_V_β_2_b and Ca_V_α_2_δ long cRNAs each. Standard two-electrode voltage clamp (TEVC) experiments were performed at room temperature (21–22 °C) in calcium-free recording solutions with barium as a charge carrier [1] containing in mM: Ba(OH)_2_ 40, NaOH 50, KOH 1, HEPES 5, pH 7.4 with methanesulfonic acid. Channels conducting barium activate much slower as calcium-dependent inactivation is abolished and inactivation is thereby nearly uniquely dependent on transmembrane voltage [35]. Niflumic acid (300 µM) was added to the solution to block Ca^2+^-activated Cl^−^ currents [36]. Currents were recorded using agar bridges 2–3 days after cRNA injection. Each oocyte was washed in four consecutive baths of recording solution prior to measurement to rinse from the ND96 solution. Microelectrodes were fabricated from glass capillary tubes and filled with 3 M KCl. Tip resistance was in the range of 0.5–1.0 MΩ. TEVC recordings were performed using a TurboTEC-10CD Amplifier (npi) with a Digidata 1200 A/D-converter (Axon Instruments, Union City, CA, USA). For data acquisition, the software pCLAMP7 (Axon Instruments) was used. The data were analyzed with ClampFit10 (Axon Instruments).

### 4.4. In Silico Heart and Beta Cell Model

In order to simulate the electrophysiological behavior of human cardiac and pancreatic beta cells, we used the human ventricular myocyte model of ten Tusscher and Panfilov [17], and the human beta cell model of Riz et al. [18], respectively. Both models are implemented in the acCELLerate software framework [37]. Ion channel gates were numerically integrated using the Rush–Larsen method [38] and all other variables using the forward Euler method with a temporal increment of 10 µs. All shown results were generated with the models being in a limit cycle, i.e., the models were simulated until none of the variables changed more than 0.1% from one beat to the next.

To represent the two mutations Ca_V_1.2^L566P^ and Ca_V_1.2^G406R^ of the Ca_V_1.2 channel, the measurement data illustrated in this work and the data published by Splawski et al. [1] were integrated. As the mutation in Timothy syndrome (Ca_V_1.2^G406R^) can be found in exon 8A, only 11.5% of Ca_V_1.2 channels express this mutation in our heterozygous representation and are combined with 88.5% of WT channels. For Ca_V_1.2^L566P^, the heterozygous model consists of 50% mutant channels and 50% WT. The mutant channel conductivity g_CaV_ for Ca_V_1.2^L566P^ was reduced to 64% to represent the loss in the peak current shown in Figure 1e. The inactivation time constant τ_h,CaV_ in the Riz et al. model is not voltage dependent but scalar. Thus, we integrated the mean of the change in time constant illustrated in Figure 2b (a difference by a factor of 2.8 between Ca_V_1.2^WT^ and Ca_V_1.2^L566P^) and changed τ_h,CaV_ from 20 ms in the WT model to 56 ms for the Ca_V_1.2^L566P^ mutation. We did not have any data concerning the change of τ_h,CaV_ for Ca_V_1.2^G406R^ thus we left its value for the mutant variant at 20 ms. The change in biophysical properties due to the mutations was integrated based on the parameters in the respective equation for the Ca_V_1.2 current:

$$I_{\mathrm{CaV}}=g_{\mathrm{CaV}}m_{\mathrm{CaV},\infty }h_{\mathrm{CaV}}\left(V_{m}-E_{\mathrm{Ca}}\right)$$

with the channel conductivity g_CaV_, the steady-state voltage-dependent activation m_CaV,∞_, the voltage-dependent inactivation h_CaV_, the transmembrane voltage V_m_, and the Nernst potential of calcium E_Ca_. h_CaV_ is calculated using the ordinary differential equation:

$$\frac{{\mathrm{dh}}_{\mathrm{CaV}}}{\mathrm{dt}}=\frac{h_{\mathrm{CaV},\infty }-h_{\mathrm{CaV}}}{\tau_{h,\mathrm{CaV}}}$$

and h_CaV,∞_ is defined by

$$h_{\mathrm{CaV},\infty }=\max\left[0,\;\min\left\{1,\;1+m_{\mathrm{CaV},\infty }\left(V_{m}-E_{\mathrm{Ca}}\right)/\phi \right\}\right]$$

with

$$m_{\mathrm{CaV},\infty }=\frac{1}{1+\exp\left(\left(V_{m}-V_{m,\mathrm{CaV}}\right)/n_{m,\;\mathrm{CaV}}\right)}$$

The parameters V_m,CaV_, n_m,CaV_, and ϕ are those representing the biophysical properties and can be adjusted so that simulated traces represent measured data. In the original model by Riz et al., V_m,CaV_ = −25 mV, n_m,CaV_ = −6 mV, and ϕ = 57 mV. The optimal values for the parameters of h_CaV,∞_ were found using an optimization framework (DOI 10.1109/IEMBS.2003.1280502) based on Powell’s optimization method to minimize for each voltage-clamp step with an increment of 10 mV the following evaluation function f:

$$f={\left(V_{1/2,\mathrm{inact},\mathrm{sim}}-V_{1/2,\mathrm{inact},\mathrm{meas}}\right)}^{2}\;\;+\overset{60\mathrm{mV}}{\underset{V_{c}=-60\mathrm{mV}}{\sum }}\left\{\frac{1}{2}{\left({\mathrm{act}}_{\mathrm{sim}}\left(V_{c}\right)-{\mathrm{act}}_{\mathrm{meas}}\left(V_{c}\right)\right)}^{2}\;\;+{\left({\mathrm{inact}}_{\mathrm{sim}}\left(V_{c}\right)-{\mathrm{inact}}_{\mathrm{meas}}\left(V_{c}\right)\right)}^{2}\right\}$$

with V_1/2,inact_ being the V_1/2_ of inactivation of Ca_V_1.2, act(V_c_) the peak current amplitude during the activation clamp protocol from −80 mV to the clamp voltage V_c_ normalized to the maximum activation current occurring in the whole activation protocol, and inact (V_c_) the peak current amplitude during the inactivation clamp protocol from the clamp voltage V_c_ to 10 mV normalized to the maximum inactivation current occurring in the whole inactivation protocol. We included the activation data into the evaluation function in order to avoid harming the activation behavior by a good fit to inactivation, as V_m,CaV_ and n_m,CaV_ change both activation and inactivation. To strengthen the influence of inactivation, we included a weighting factor of 1/2 to the activation component. The parameter range of optimization was limited to [−100 mV:100 mV] for V_m,CaV_, to [−20 mV:0 mV] for n_m,CaV_, and [0 mV:1000 mV] for ϕ.

The resulting parameters for Ca_V_1.2^L566P^ are V_h,CaV_ = −21.61 mV, n_h,CaV_ = −5.69 mV, and ϕ = 66.84 mV and for Ca_V_1.2^G406R^ they are V_h,CaV_ = −18.54 mV, n_h,CaV_ = −7.36 mV, and ϕ = 243.01 mV. Figure S1 shows the resulting voltage dependence of the inactivation of currents in cardiomyocytes using the ten Tusscher and Panfilov models for Ca_V_1.2^WT^, Ca_V_1.2^L566P^, and Ca_V_1.2^G406R^. Figure S2 depicts the same using the beta cell model of Riz et al.

To analyze membrane oscillations in Figure 5C,D, n_mCaPQ_ was set from −6 mV to −10 mV as in the original Riz et al. model, the maximum SK channel conductivity g_SK_ from 0.1 nS/pF to 0.035 nS/pF, and the maximum Kv channel conductivity g_Kv_ from 1 nS/pF to 0.24 nS/pF.

### 4.5. Retrospective Chart Record of TS Patients Reporting Hypoglycemia

From the Timothy Syndrome Foundation Registry, *n* = 18 TS patients were retrospectively assessed by one of the authors for the presence of hypoglycemia at any time in their patient history (K.T.). Of these, five patients had reported hypoglycemic episodes in the past and were asked to provide medical records and/or any other information about these hypoglycemic events for retrospective review. As the initial reports that became available to us already demonstrated severe hypoglycemia-associated morbidity and even mortality, we judged formal structured clinical evaluation for hypoglycemia, including provocative fasting studies for these patients, only acceptable if strictly clinically indicated, as hypoglycemia was associated with sudden cardiac deterioration in some and even fatality in two patients. Later, we learned about one further TS patient who underwent metabolic evaluation at the age of two years after having experienced hypoglycemia aged 9 months old (data presented in Table S4). All patients or their respective guardians gave informed consent to be part of this work.

### 4.6. Statistics

For the statistical analysis of TEVC experiments, pClamp 10.0 (Molecular Devices, San Jose, CA, USA) and Origin 2016 (OriginLab Corporation, Northampton, MA, United States of America) software were used. All fitting procedures were based on the simplex algorithm. Voltage dependence of inactivation was analyzed by fitting to a Boltzmann equation and rate of inactivation was analyzed with mono-exponential fits. The results are reported as mean ± s.e.m. (*n* = number of individual patches or oocytes). Significance was probed using an unpaired two-tailed Student’s *t*-test with a *p* value of *p* < 0.05 (*), *p* < 0.01 (**) and *p* < 0.001 (***).

### 4.7. Ethics

The study was conducted in accordance with the Declaration of Helsinki in the latest revision of 2013, and its prospective part was approved by the Ethics Committee of the Medical Faculty of the Heinrich-Heine University Duesseldorf (ID 4790R, approved on 31.10.2014). The animal study using *Xenopus* toads was approved by the Ethics Committee of the Regierungspräsidium Giessen (protocol code V54-19c 20 15 h 02 MR 20/28 Nr.A 23/2017, approved on 12 February 2018). Informed consent was obtained from all subjects involved in the study.

## Data Availability

The data used to support the findings of this study are included in the article.

## Supplementary Materials

The following supporting information can be downloaded at: https://www.mdpi.com/article/10.3390/ijms23158097/s1.

## References

[1] Splawski I., Timothy K.W., Sharpe L.M., Decher N., Kumar P., Bloise R., Napolitano C., Schwartz P.J., Joseph R.M., Condouris K. (2004). Ca_V_1.2 calcium channel dysfunction causes a multisystem disorder including arrhythmia and autism. Cell.

[2] Yang S.N., Berggren P.O. (2006). The role of voltage-gated calcium channels in pancreatic beta-cell physiology and pathophysiology. Endocr. Rev..

[3] Schulla V., Renstrom E., Feil R., Feil S., Franklin I., Gjinovci A., Jing X.J., Laux D., Lundquist I., Magnuson M.A. (2003). Impaired insulin secretion and glucose tolerance in beta cell-selective Ca_V_1.2 Ca^2+^ channel null mice. EMBO J..

[4] Tuluc P., Theiner T., Jacobo-Piqueras N., Geisler S.M. (2021). Role of High Voltage-Gated Ca^2+^ Channel Subunits in Pancreatic beta-Cell Insulin Release. From Structure to Function. Cells.

[5] Reinbothe T.M., Alkayyali S., Ahlqvist E., Tuomi T., Isomaa B., Lyssenko V., Renstrom E. (2013). The human L-type calcium channel Cav1.3 regulates insulin release and polymorphisms in *CACNA1D* associate with type 2 diabetes. Diabetologia.

[6] Bruning G.J. (1990). Recent advances in hyperinsulinism and the pathogenesis of diabetes mellitus. Curr. Opin. Peadiatr..

[7] Otonkoski T., Ammälä C., Huopio H., Cote G.J., Chapman J., Cosgrove K., Ashfield R., Huang E., Komulainen J., Ashcroft F.M. (1999). A point mutation inactivating the sulfonylurea receptor causes the severe form of persistent hyperinsulinemic hypoglycemia of infancy in Finland. Diabetes.

[8] Yau D., Laver T.W., Dastamani A., Senniappan S., Houghton J.A.L., Shaikh G., Cheetham T., Mushtaq T., Kapoor R.R., Randell T. (2020). Using referral rates for genetic testing to determine the incidence of a rare disease: The minimal incidence of congenital hyperinsulinism in the UK is 1 in 28,389. PLoS ONE.

[9] Helleskov A., Melikyan M., Globa E., Shcherderkina I., Poertner F., Larsen A.M., Filipsen K., Brusgaard K., Christiansen C.D., Hansen L.K. (2017). Both Low Blood Glucose and Insufficient Treatment Confer Risk of Neurodevelopmental Impairment in Congenital Hyperinsulinism: A Multinational Cohort Study. Front. Endocrinol..

[10] Muukkonen L., Männistö J., Jääskeläinen J., Hannonen R., Huopio H. (2019). The effect of hypoglycaemia on neurocognitive outcome in children and adolescents with transient or persistent congenital hyperinsulinism. Dev. Med. Child Neurol..

[11] Stanley C.A. (2016). Perspective on the Genetics and Diagnosis of Congenital Hyperinsulinism Disorders. J. Clin. Endocrinol. Metab..

[12] Kapoor R.R., Flanagan S.E., Arya V.B., Shield J.P., Ellard S., Hussain K. (2013). Clinical and molecular characterisation of 300 patients with congenital hyperinsulinism. Eur. J. Endocrinol..

[13] Snider K.E., Becker S., Boyajian L., Shyng S.L., MacMullen C., Hughes N., Ganapathy K., Bhatti T., Stanley C.A., Ganguly A. (2013). Genotype and phenotype correlations in 417 children with congenital hyperinsulinism. J. Clin. Endocrinol. Metab..

[14] Cifaldi C., Brigida I., Barzaghi F., Zoccolillo M., Ferradini V., Petricone D., Cicalese M.P., Lazarevic D., Cittaro D., Omrani M. (2019). Targeted NGS Platforms for genetic screening and gene discovery in primary immunodeficiencies. Front. Immunol..

[15] Ponzi E., Maiorana A., Lepri F.R., Mucciolo M., Semeraro M., Taurisano R., Olivieri G., Novelli A., Dionisi-Vici C. (2018). Persistent Hypoglycemia in Children: Targeted Gene Panel Improves the Diagnosis of Hypoglycemia Due to Inborn Errors of Metabolism. J. Pediatr..

[16] Schwartz P.J., Moss A.J., Vincent G.M., Crampton R.S. (1993). Diagnostic criteria for the long QT syndrome. An update. Circulation.

[17] Ten Tusscher K.H., Panfilov A.V. (2006). Alternans and spiral breakup in a human ventricular tissue model. Am. J. Physiol. Heart Circ. Physiol..

[18] Riz M., Braun M., Pedersen M.G. (2014). Mathematical modeling of heterogeneous electrophysiological responses in human beta-cells. PLoS Comput. Biol..

[19] Rorsman P., Braun M. (2013). Regulation of insulin secretion in human pancreatic islets. Annu. Rev. Physiol..

[20] De Mingo Alemany M.C., Mifsud Grau L., Moreno Macián F., Ferrer Lorente B., León Cariñena S. (2020). A de novo *CACNA1D* missense mutation in a patient with congenital hyperinsulinism, primary hyperaldosteronism and hypotonia. Channels.

[21] Flanagan S.E., Vairo F., Johnson M.B., Caswell R., Laver T.W., Lango Allen H., Hussain K., Ellard S. (2017). A *CACNA1D* mutation in a patient with persistent hyperinsulinaemic hypoglycaemia, heart defects, and severe hypotonia. Pediatr. Diabetes.

[22] Torekov S.S., Iepsen E., Christiansen M., Linneberg A., Pedersen O., Holst J.J., Kanters J.K., Hansen T. (2014). KCNQ1 long QT syndrome patients have hyperinsulinemia and symptomatic hypoglycemia. Diabetes.

[23] Varró A., Tomek J., Nagy N., Virág L., Passini E., Rodriguez B., Baczkó I. (2021). Cardiac transmembrane ion channels and action potentials: Cellular physiology and arrhythmogenic behavior. Physiol. Rev..

[24] Rorsman P., Ashcroft F.M. (2018). Pancreatic beta-Cell Electrical Activity and Insulin Secretion: Of Mice and Men. Physiol. Rev..

[25] Abernethy D.R., Soldatov N.M. (2002). Structure-functional diversity of human L-type Ca^2+^ channel: Perspectives for new pharmacological targets. J. Pharmacol. Exp. Ther..

[26] Wielowieyski P.A., Wigle J.T., Salih M., Hum P., Tuana B.S. (2001). Alternative splicing in intracellular loop connecting domains II and III of the alpha 1 subunit of Ca_V_1.2 Ca^2+^ channels predicts two-domain polypeptides with unique C-terminal tails. J. Biol. Chem..

[27] Etheridge S.P., Bowles N.E., Arrington C.B., Pilcher T., Rope A., Wilde A.A., Alders M., Saarel E.V., Tavernier R., Timothy K.W. (2011). Somatic mosaicism contributes to phenotypic variation in Timothy syndrome. Am. J. Med. Genet. Part A.

[28] Baurand A., Falcon-Eicher S., Laurent G., Villain E., Bonnet C., Thauvin-Robinet C., Jacquot C., Eicher J.C., Gourraud J.B., Schmitt S. (2017). Incomplete Timothy syndrome secondary to a mosaic mutation of the *CACNA1C* gene diagnosed using next-generation sequencing. Am. J. Med. Genet. Part A.

[29] Eichmann D., Hufnagel M., Quick P., Santer R. (1999). Treatment of hyperinsulinaemic hypoglycaemia with nifedipine. Eur. J. Pediatr..

[30] Khawash P., Hussain K., Flanagan S.E., Chatterjee S., Basak D. (2015). Nifedipine in Congenital Hyperinsulinism-A Case Report. J. Clin. Res. Pediatric Endocrinol..

[31] Güemes M., Shah P., Silvera S., Morgan K., Gilbert C., Hinchey L., Hussain K. (2017). Assessment of Nifedipine Therapy in Hyperinsulinemic Hypoglycemia due to Mutations in the ABCC8 Gene. J. Clin. Endocrinol. Metab..

[32] Koklu E., Ozkan K.U., Sayar H., Köklü S., Keskin M. (2013). Treatment of hyperinsulinemic hypoglycemia because of diffuse nesidioblastosis with nifedipine after surgical therapies in a newborn. J. Pediatr. Endocrinol. Metab..

[33] Richards S., Aziz N., Bale S., Bick D., Das S., Gastier-Foster J., Grody W.W., Hegde M., Lyon E., Spector E. (2015). Standards and guidelines for the interpretation of sequence variants: A joint consensus recommendation of the American College of Medical Genetics and Genomics and the Association for Molecular Pathology. Genet. Med..

[34] Streit A.K., Netter M.F., Kempf F., Walecki M., Rinné S., Bollepalli M.K., Preisig-Müller R., Renigunta V., Daut J., Baukrowitz T. (2011). A specific two-pore domain potassium channel blocker defines the structure of the TASK-1 open pore. J. Biol. Chem..

[35] Lee K.S., Marban E., Tsien R.W. (1985). Inactivation of calcium channels in mammalian heart cells: Joint dependence on membrane potential and intracellular calcium. J. Physiol..

[36] White M.M., Aylwin M. (1990). Niflumic and flufenamic acids are potent reversible blockers of Ca^2+^-activated Cl^−^ channels in *Xenopus* oocytes. Mol. Pharmacol..

[37] Seemann G., Sachse F.B., Karl M., Weiss D.L., Heuveline V., Doessel O., Fitt A., Norbury J., Ockendon H., Wilson E. (2010). Framework for Modular, Flexible and Efficient Solving the Cardiac Bidomain Equations Using PETSc. Progress in Industrial Mathematics at ECMI 2008.

[38] Rush S., Larsen H. (1978). A practical algorithm for solving dynamic membrane equations. IEEE Trans. Biomed. Eng..

