# Supplementary material for: Hyperinsulinemic Hypoglycemia Associated with a CaV1.2 Variant with Mixed Gain- and Loss-of-Function Effects
Source: Int J Mol Sci. 2022 Jul 22;23(15):8097. doi: 10.3390/ijms23158097 (PMC9332183; doi:10.3390/ijms23158097)
Supplement: Supplementary file 1 [file ijms-23-08097-s001.zip › Supplemental Figures 2022-06-30 FINAL.pdf]

# Figure S1

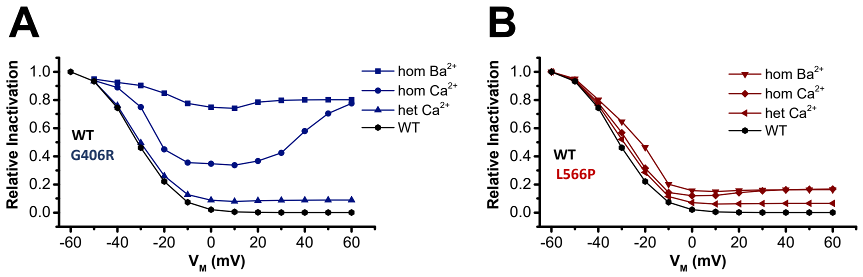


**Supplementary Figure S1.** Model of the voltage-dependence of inactivation of currents in cardiomyocytes using the ten Tusscher model. (**a**) Voltage-dependence of inactivation for $Ca_V1.2^{G406R}$ (blue) and $Ca_V1.2^{WT}$ (black) with $Ca^{2+}$ or $Ba^{2+}$ as charge carrier (hom, 100%, homozygous, het, 11.5%, heterozygous). $V_M$, membrane voltage. (**b**) Voltage dependence of inactivation for $Ca_V1.2^{L566P}$ (red) and $Ca_V1.2^{WT}$ (black) with $Ca^{2+}$ or $Ba^{2+}$ as charge carrier in the homozygous (hom, 100%) or heterozygous state (het, 50%) as indicated. $V_M$, membrane voltage.

# Figure S2

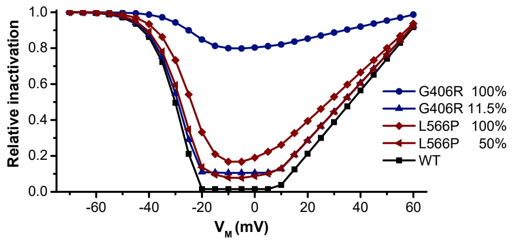


**Supplementary Figure S2.** Model of the voltage-dependence of inactivation of calcium currents in ß-cells calculated using the Riz model. Voltage-dependence of inactivation for $Ca_V1.2^{L566P}$ (red), $Ca_V1.2^{G406R}$ (blue) and $Ca_V1.2^{WT}$ (black) in the homozygous or heterozygous state as indicated. $V_M$, membrane voltage.
