# Supplementary material for: Hyperinsulinemic Hypoglycemia Associated with a CaV1.2 Variant with Mixed Gain- and Loss-of-Function Effects
Source: Int J Mol Sci. 2022 Jul 22;23(15):8097. doi: 10.3390/ijms23158097 (PMC9332183; doi:10.3390/ijms23158097)
Supplement: Supplementary file 1 [file ijms-23-08097-s001.zip › Supplementary Table S1.pdf]

Supplementary Table S1. Analyzed genes in NGS panel, total n=219.

Gene Symbol	% Nuc. Min. 15X Cov.	Coding Exons Min. 15X Cov. / All Coding Exons	Coding Exons Not Min. 15X Cov.	Coding Exons Min. 15X Cov.
ABCB11	100.00%	27 / 27	None	All
ABCC8	100.00%	39 / 39	None	All
ACADM	100.00%	13 / 13	None	All
ACADS	100.00%	11 / 11	None	All
ACADVL	100.00%	22 / 22	None	All
ACAT1	100.00%	12 / 12	None	All
ADCY5	99.21%	21 / 22	1	2, 3, 4, 5, 6, 7, 8, 9, 10, 11, 12, 13, 14, 15, 16, 17, 18, 19, 20, 21, 22
ADIPOQ	100.00%	2 / 2	None	All
AGL	100.00%	34 / 34	None	All
AGMO	100.00%	13 / 13	None	All
AGPAT2	100.00%	6 / 6	None	All
AKT2	100.00%	13 / 13	None	All
ALDH7A1	100.00%	18 / 18	None	All
ALDOB	100.00%	8 / 8	None	All
ALG3	100.00%	10 / 10	None	All
ALMS1	100.00%	23 / 23	None	All
ARAP1	100.00%	33 / 33	None	All
ARL6	100.00%	7 / 7	None	All
BAD	100.00%	3 / 3	None	All
BBS1	100.00%	17 / 17	None	All
BBS10	100.00%	2 / 2	None	All
BBS12	100.00%	1 / 1	None	All
BBS2	100.00%	17 / 17	None	All
BBS4	100.00%	16 / 16	None	All
BBS5	100.00%	12 / 12	None	All
BBS7	100.00%	19 / 19	None	All
BBS9	99.70%	21 / 22	7	2, 3, 4, 5, 6, 8, 9, 10, 11, 12, 13, 14, 15, 16, 17, 18, 19, 20, 21, 22, 23
BCS1L	100.00%	7 / 7	None	All
BDNF	100.00%	5 / 5	None	All
BLK	100.00%	12 / 12	None	All
BSCL2	100.00%	11 / 11	None	All
C10orf2	100.00%	5 / 5	None	All
C19orf80	100.00%	4 / 4	None	All
CACNA1C	100.00%	52 / 52	None	All
CAPN10	100.00%	12 / 12	None	All
CAV1	100.00%	3 / 3	None	All
CD36	100.00%	12 / 12	None	All
CDK4	100.00%	7 / 7	None	All
CEL	96.47%	10 / 11	11	1, 2, 3, 4, 5, 6, 7, 8, 9, 10

CEP290	99.92%	52 / 53	7	2, 3, 4, 5, 6, 8, 9, 10, 11, 12, 13, 14, 15, 16, 17, 18, 19, 20, 21, 22, 23, 24, 25, 26, 27, 28, 29, 30, 31, 32, 33, 34, 35, 36, 37, 38, 39, 40, 41, 42, 43, 44, 45, 46, 47, 48, 49, 50, 51, 52, 53, 54
CHGA	97.35%	7 / 8	1	2, 3, 4, 5, 6, 7, 8
CHGB	100.00%	5 / 5	None	All
CIDEC	100.00%	7 / 7	None	All
CISD2	100.00%	3 / 3	None	All
CPT1A	100.00%	19 / 19	None	All
CPT2	100.00%	5 / 5	None	All
CYC1	85.71%	6 / 7	1	2, 3, 4, 5, 6, 7
CYP11A1	100.00%	9 / 9	None	All
DGUOK	100.00%	7 / 7	None	All
EIF2AK3	97.54%	16 / 17	1	2, 3, 4, 5, 6, 7, 8, 9, 10, 11, 12, 13, 14, 15, 16, 17
ETFA	100.00%	12 / 12	None	All
ETFB	100.00%	6 / 6	None	All
ETFDH	100.00%	13 / 13	None	All
FBN1	100.00%	65 / 65	None	All
FBP1	100.00%	7 / 7	None	All
FGF8	83.33%	5 / 6	3	4, 5, 6, 7, 8
FGFR1	100.00%	19 / 19	None	All
FOXA1	100.00%	2 / 2	None	All
FOXA2	100.00%	2 / 2	None	All
FOXA3	100.00%	2 / 2	None	All
FOXO1	81.23%	1 / 2	1	2
FOXP3	100.00%	11 / 11	None	All
FXN	93.42%	5 / 6	1	2, 3, 4, 5, 6
G6PC	100.00%	5 / 5	None	All
GALE	100.00%	10 / 10	None	All
GALK1	100.00%	8 / 8	None	All
GALT	100.00%	11 / 11	None	All
GATA4	91.14%	5 / 6	2	3, 4, 5, 6, 7
GATA6	96.88%	5 / 6	2	3, 4, 5, 6, 7
GBE1	100.00%	16 / 16	None	All
GCG	100.00%	5 / 5	None	All
GCGR	100.00%	13 / 13	None	All
GCK	100.00%	12 / 12	None	All
GCKR	100.00%	19 / 19	None	All
GFM1	100.00%	18 / 18	None	All
GH1	100.00%	5 / 5	None	All
GHR	100.00%	10 / 10	None	All
GHRHR	100.00%	13 / 13	None	All

GK	100.00%	21 / 21	None	All
GLI2	99.00%	12 / 13	13	1, 2, 3, 4, 5, 6, 7, 8, 9, 10, 11, 12
GLIS3	100.00%	10 / 10	None	All
GLP1R	100.00%	13 / 13	None	All
GLUD1	100.00%	13 / 13	None	All
GYS1	100.00%	16 / 16	None	All
GYS2	100.00%	16 / 16	None	All
HADH	100.00%	9 / 9	None	All
HADHA	100.00%	20 / 20	None	All
HADHB	100.00%	16 / 16	None	All
HESX1	100.00%	4 / 4	None	All
HFE	100.00%	7 / 7	None	All
HMGCL	100.00%	9 / 9	None	All
HMGCS2	100.00%	9 / 9	None	All
HNF1A	100.00%	10 / 10	None	All
HNF1B	100.00%	9 / 9	None	All
HNF4A	100.00%	13 / 13	None	All
HYMAI	94.79%	0 / 2	All	None
IER3IP1	100.00%	3 / 3	None	All
IGF1	100.00%	6 / 6	None	All
IGF1R	100.00%	21 / 21	None	All
IGFALS	100.00%	2 / 2	None	All
INS	100.00%	2 / 2	None	All
INSR	95.45%	21 / 22	1	2, 3, 4, 5, 6, 7, 8, 9, 10, 11, 12, 13, 14, 15, 16, 17, 18, 19, 20, 21, 22
ISL1	100.00%	6 / 6	None	All
KCNJ11	100.00%	1 / 1	None	All
KCNQ1	97.03%	16 / 17	1	2, 3, 4, 5, 6, 7, 8, 9, 10, 11, 12, 13, 14, 15, 16, 17
KDM6A	100.00%	30 / 30	None	All
KLF11	100.00%	4 / 4	None	All
KLF14	76.81%	0 / 1	All	None
KMT2D	100.00%	54 / 54	None	All
LEP	100.00%	2 / 2	None	All
LEPR	95.24%	20 / 21	21	3, 4, 5, 6, 7, 8, 9, 10, 11, 12, 13, 14, 15, 16, 17, 18, 19, 20, 22, 23
LHX3	97.49%	6 / 7	2	1, 3, 4, 5, 6, 7
LHX4	100.00%	6 / 6	None	All
LMNA	100.00%	15 / 15	None	All
LMNB2	96.95%	11 / 12	1	2, 3, 4, 5, 6, 7, 8, 9, 10, 11, 12
MADD	100.00%	35 / 35	None	All
MAFA	82.26%	0 / 1	All	None
MAFB	100.00%	1 / 1	None	All
MBD5	100.00%	10 / 10	None	All

MC1R	100.00%	1 / 1	None	All
MC2R	100.00%	1 / 1	None	All
MC4R	100.00%	1 / 1	None	All
MCCC1	100.00%	19 / 19	None	All
MCCC2	100.00%	17 / 17	None	All
MCM4	98.96%	15 / 16	2	3, 4, 5, 6, 7, 8, 9, 10, 11, 12, 13, 14, 15, 16, 17
MEF2C	100.00%	11 / 11	None	All
MKKS	100.00%	4 / 4	None	All
MKS1	100.00%	19 / 19	None	All
MNX1	86.88%	3 / 4	1	2, 3, 4
MPC1	100.00%	4 / 4	None	All
MPC2	100.00%	5 / 5	None	All
MPI	100.00%	8 / 8	None	All
MPV17	100.00%	7 / 7	None	All
MRAP	100.00%	4 / 4	None	All
MST1	100.00%	18 / 18	None	All
MTNR1B	100.00%	2 / 2	None	All
MYT1	100.00%	21 / 21	None	All
NEUROD1	100.00%	1 / 1	None	All
NEUROG3	100.00%	1 / 1	None	All
NKX2-2	100.00%	2 / 2	None	All
NKX6-1	96.89%	1 / 3	1, 3	2
NNT	100.00%	21 / 21	None	All
NOS1AP	100.00%	10 / 10	None	All
NOTCH2	99.40%	33 / 34	1	2, 3, 4, 5, 6, 7, 8, 9, 10, 11, 12, 13, 14, 15, 16, 17, 18, 19, 20, 21, 22, 23, 24, 25, 26, 27, 28, 29, 30, 31, 32, 33, 34
NR0B1	100.00%	2 / 2	None	All
NR3C1	100.00%	9 / 9	None	All
NTRK2	100.00%	20 / 20	None	All
OXCT1	100.00%	17 / 17	None	All
PAX4	100.00%	9 / 9	None	All
PAX6	100.00%	11 / 11	None	All
PBX1	100.00%	10 / 10	None	All
PCK1	100.00%	9 / 9	None	All
PCK2	100.00%	10 / 10	None	All
PCSK1	100.00%	15 / 15	None	All
PDX1	100.00%	2 / 2	None	All
PFKFB2	100.00%	15 / 15	None	All
PFKFB3	94.12%	16 / 17	1	2, 3, 4, 5, 6, 7, 8, 9, 10, 11, 12, 13, 14, 15, 16, 17
PGM1	100.00%	12 / 12	None	All
PHKA1	100.00%	32 / 32	None	All

PHKA2	100.00%	33 / 33	None	All
PHKB	100.00%	33 / 33	None	All
PHKG2	100.00%	10 / 10	None	All
PHOX2B	95.40%	2 / 3	3	1, 2
PLAGL1	100.00%	2 / 2	None	All
PMM1	100.00%	8 / 8	None	All
PMM2	100.00%	8 / 8	None	All
POLG	100.00%	22 / 22	None	All
POMC	100.00%	2 / 2	None	All
POU1F1	100.00%	6 / 6	None	All
PPARA	100.00%	6 / 6	None	All
PPARG	100.00%	7 / 7	None	All
PRKAG2	100.00%	16 / 16	None	All
PRKAG3	100.00%	13 / 13	None	All
PROKR2	100.00%	2 / 2	None	All
PROP1	100.00%	3 / 3	None	All
PROX1	100.00%	4 / 4	None	All
PTEN	100.00%	9 / 9	None	All
PTF1A	88.81%	1 / 2	1	2
PTRF	100.00%	2 / 2	None	All
PYGL	100.00%	20 / 20	None	All
RET	100.00%	20 / 20	None	All
RFX1	98.76%	19 / 20	21	2, 3, 4, 5, 6, 7, 8, 9, 10, 11, 12, 13, 14, 15, 16, 17, 18, 19, 20
RFX6	100.00%	19 / 19	None	All
SDCCAG8	100.00%	18 / 18	None	All
SEL1L	100.00%	21 / 21	None	All
SH2B1	100.00%	9 / 9	None	All
SLC16A1	100.00%	4 / 4	None	All
SLC19A2	100.00%	6 / 6	None	All
SLC22A5	100.00%	10 / 10	None	All
SLC25A13	100.00%	18 / 18	None	All
SLC25A20	100.00%	9 / 9	None	All
SLC2A1	100.00%	10 / 10	None	All
SLC2A2	100.00%	11 / 11	None	All
SLC30A8	100.00%	8 / 8	None	All
SLC37A4	100.00%	10 / 10	None	All
SOX3	96.03%	0 / 1	All	None
SOX4	89.13%	0 / 1	All	None
STAR	100.00%	7 / 7	None	All
STAT5B	100.00%	18 / 18	None	All
SUCLG1	88.89%	8 / 9	9	1, 2, 3, 4, 5, 6, 7, 8
SUMO1	100.00%	5 / 5	None	All
TALDO1	100.00%	8 / 8	None	All
TAZ	100.00%	11 / 11	None	All
TCF7L2	100.00%	18 / 18	None	All

TP53INP1	100.00%	4 / 4	None	All
TRIM32	100.00%	1 / 1	None	All
TRMU	100.00%	11 / 11	None	All
TTC8	100.00%	17 / 17	None	All
UCP2	100.00%	6 / 6	None	All
UQCRB	100.00%	5 / 5	None	All
UQCRC2	100.00%	14 / 14	None	All
VPS13C	100.00%	86 / 86	None	All
WDPCP	94.74%	18 / 19	8	1, 2, 3, 4, 5, 6, 7, 9, 10, 11, 12, 13, 14, 15, 16, 17, 18, 19
WFS1	100.00%	7 / 7	None	All
WRN	100.00%	34 / 34	None	All
YARS2	100.00%	5 / 5	None	All
YY1	100.00%	5 / 5	None	All
ZFP57	100.00%	4 / 4	None	All
ZMPSTE24	100.00%	10 / 10	None	All
