# Supplementary material for: Hyperinsulinemic Hypoglycemia Associated with a CaV1.2 Variant with Mixed Gain- and Loss-of-Function Effects
Source: Int J Mol Sci. 2022 Jul 22;23(15):8097. doi: 10.3390/ijms23158097 (PMC9332183; doi:10.3390/ijms23158097)
Supplement: Supplementary file 1 [file ijms-23-08097-s001.zip › Supplementary Table S2.pdf]

Supplementary Table S2. Variants of unknown significance.

	Isoform	Coding DNA	Protein	Zygosity	rs#	Clin assoc.	PubMed ID	Funct. Pred	Cons. Pred.	% covered	Comment
ALMS1	NM_015120.4	c.4344dupT	p.Pro1448fs	het.		-	-	-	-	100	heterozygous variant in autosomal recessive disease
ETFB	NM_001985.2	c.152G>A	p.Arg51Gln	het.		-	-	6/8	4/6	100	heterozygous variant in autosomal recessive disease
