# Supplementary material for: Hyperinsulinemic Hypoglycemia Associated with a CaV1.2 Variant with Mixed Gain- and Loss-of-Function Effects
Source: Int J Mol Sci. 2022 Jul 22;23(15):8097. doi: 10.3390/ijms23158097 (PMC9332183; doi:10.3390/ijms23158097)
Supplement: Supplementary file 1 [file ijms-23-08097-s001.zip › Supplementary Table S3.pdf]

Supplementary Table S3. Retrospective chart record analysis of TS patients with reported episodes of hypoglycemia.

Pat. Nr	*CACNA1C* sequence variant	Cardiac phenotype	Further syndromic features	Age at reported hypo-glycemia	Hypoglycemia -predisposing conditions	Medication at the time of hypoglycemia	Symptoms at time of hypoglycemia	Available metabolic results at the time of hypoglycemia	Treatment/outcome of hypoglycemic episode	Previous episodes suggestive of hypoglycemia	Suspected reason for hypoglycemia in medical reports, given recommendations	subsequent hypoglycemia
1, female	G406R Exon 8A	LQT, Manifestation:Cardiac arrest aged 10m under general anesthesia No ICD	Ilieal atresia, left hip dysplasia, bilateral 3-5 finger syndactyly, bilateral 1-5 toe syndactyly, mild micrognathia, low set ears, broad forehead, thin upper lip, mandibular proganthia, small stature, physical delays	11m	First night home after discharge from hip surgery, overnight fast	Spironolactone 0,8ml/d, propranolol 0,71ml 3x/d	Sudden loss of consciousness, pale, both pupils reactive, HR 62/min, RR 34/min RR 108/56mmHg	BG 29mg/dl, otherwise normal basic clinical chemistry, however no metabolic profile performed	i.v. dextrose and i.v. glucagon 20 µg/kg simultaneously, raised BG to 86mg/dl, complete resolution of symptoms	Yes, during previous surgery	Beta blocker overdose	No
2, male	G406R Exon 8A	LQT, Manifestation: 2:1 heart block under general anesthesia No ICD	Bilateral 3-5 finger syndactyly, Bilateral 1-5 toe syndactyly, low set ears, broad forehead, mandibular proganthia, mild physical and mental delays	1y 8m	Hypoglycemia before breakfast after overnight fast for ~11h, not different from other nights before.	propranolol	Stupor, very difficult to arouse, intermittent myoclonic jerks/seizure. HR 60-65/min. Pale, diaphoretic. Heart monitor without cardiac arrythmias	BG 37mg/dl otherwise normal basic clinical chemistry, however no metabolic profile performed	i.v. dextrose → complete resolution of hypoglycemic symptoms	Episode of muscle weakness 1m before, suspected to be periodic paralysis	Beta blocker side effect Avoid fasting for >8h, bedtime snack.	No
3, male	G406R Exon 8A	LQT Diagnosis by screening ECG,	Bilateral 3-5 finger syndactyly, bilateral 2-3	3y	Parents report exhausting museum visit the day before	Inhalative budesonide, mometasone nasal spray,	Hypoglycemic seizure, respiratory	BG <20mg/dl, urine dipstick with only traces of ketones (15mg/dl),	i.v. dextrose → complete resolution of	None	Insulin-mediated hypoglycemia suspected from	Subsequent hypoglycemia 5yo

		ICD placed prophylactically, sympathectomy	toe syndactyly, minor mandibular proganthia, low set ears, broad forehead, small stature, mental delays small stature (length -3SDS)		and possible concomitant infection, hypoglycemia before breakfast, no clear overnight fast	nadolol 20mg/d	failure, delayed milestones	respiratory acidosis, no further metabolic workup performed	hypoglycemic symptoms		low-ketones in hypoglycemia. Avoid fasting for >12h, snack at bedtime; glucose and glucagon when hypoglycemia recurs. BG measurements in the morning. Critical sample should be obtained at the time of hypoglycemia	after overnight fast and refused breakfast led to hypoglycemic seizure with subsequent severe cardiac deterioration, finally ending in multiorgan failure and death
4, female	G406R Exon 8	LQT, diagnosis after birth due to bradycardia, pacemaker placed due t AV block 1mo; ICD placed 3yo	Neonatal seizures, developmental delay, prematurity (33w GA), failure to thrive, sensineural hearing loss, optical nerve hypoplasia no syndactyly, wide nasal bridge, mild esotropia, broad forehead, thin upper lip, low set ears, small stature, optic nerve hypoplasia	3y	Fever with decreased oral intake since the day before, hypo early morning	propranolol, phenobarbitale, budesonide	Hypoglycemic seizure, subsequent torsades de pointes and cardiac arrest after correction of hypoglycemia	After treatment of hypoglycemia BG 225mg/dl Urine ketones reported as 40mg/dl (method of determination not documented)	Glucagon (rose BG from udetectable to 22mg/dl), i.v. glucose via i.o. access. Further hypoglycemia during PICU stay, switched to p.o. glucose later	Glucose down to the 22-31 range initially after birth, but subsequently corrected. Home BG measurements performed	Poor oral intake, propranolole, "associated with Timothy syndrome by unknown pathophysiology" "Consider checking with endocrinology and/or genetic/metabolic consultation about strategies for managing hypoglycemic episodes"	Occasion of suspected mild hypoglycemia, but not warranting any therapeutic attenction
5, male	G406R Exon 8A	LQT, small PDA and PFO, sympathectomy, and ICD	Bilateral 4-5 finger syndactyly, bilateral 2-3 toe syndactyly, flat bridge	6y	Hypoglycemia after surgery, unclear if there was glucose-containing infusion during fasting period	Acetaminophen	Torsade de pointes after dental surgery	BG initially checked after surgery when cardiac detoriation occurred: 13mg/dl. no metabolic profile performed	i.v. glucose, cardiac detoriation, multiple Torsades, later multi organ	about six times in retrospect hypoglycemia is suspected. Could not arouse, cyanosis, home		Passed away

			nose, low set ears, myopia, low muscle tone, autism, physical and social delays, kidney reflux, diseased teeth, small stature						failure leading to death	rhythm strip detected no arrhythmias; when hospitalized on occasion no arrhythmia found. No seizures, no blood sugar monitoring done. Reported hypoglycemia at birth, bradycardic and in 2:1AV-block, blue and stopped breathing.		
