# Supplementary material for: Hyperinsulinemic Hypoglycemia Associated with a CaV1.2 Variant with Mixed Gain- and Loss-of-Function Effects
Source: Int J Mol Sci. 2022 Jul 22;23(15):8097. doi: 10.3390/ijms23158097 (PMC9332183; doi:10.3390/ijms23158097)
Supplement: Supplementary file 1 [file ijms-23-08097-s001.zip › Supplementary Table S4.pdf]

Supplementary Table S4. Endocrine/metabolic workup of a patient with TS syndrome at the age of two years, after a history of sporadic hypoglycemia during an episode of gastroenteritis in the age of nine months.

Patient characteristics: male G406R exon 8A; Cardiac Phenotype: LQT, 2:1 heart block under general anesthesia, no ICD at time of surgery, currently Bilateral sympathectomy and ICD; Further syndrome features: Bilateral 3-5 finger syndactyly, bilateral 2-3 toe syndactyly, low set ears, broad forehead, hypotonia; Medications at time of hypoglycemia: propranolol, mexiletine; Symptoms at time of hypoglycemia: weak, pale coloring; Previous episodes suggesting hypoglycemia: none prior to evaluation
Suspected reason for hypoglycemia: hypoglycemic death recognized in other TS children
Subsequent hypoglycemia during stress with preoperative peripheral venous line for right denervation after 8 hour fasting.

Glucose tolerance test (1,75g Glucose per kg body weight)

	Plasma glucose
Baseline (0min)	83mg/dl (4.6mmol/l)
60min	175mg/dl (9.7mmol/l)
90min	140mg/dl (7.8mmol/l)
120min	133mg/dl (7.4mmol/l)
150min	53mg/dl (2.9mmol/l)
180min	62mg/dl (3.4mmol/l)

Fasting test:
Critical sample at the end of the test (19h)

Plasma Glucose	61mg/dl (3.4mmol/l)
AST	36U/l
ALT	18U/l
Ammonia	21.6µmol/l
Free Fatty Acids	1.94mmol/l
Lactic Acid Level	1.44mmol/l
Random Cortisol	9.71µg/dl
Human Growth Hormone	6.96ng/ml
Beta-Hydroxybutyric Acid	3.67mmol/l

Low-dose Cosyntropin stimulation test (1µg Cosyntropin)

Baseline	19.91µg/dl
30min	24.5µg/dl
60min	30.35µg/dl
